# The effects of different routes of inulin administration on gut microbiota and survival rate of Indian white shrimp post-larvae (*Fenneropenaeus indicus*)

**Published:** 2015-12-15

**Authors:** Seyed Hossein Hoseinifar, Parviz Zare, Hamed Kolangi Miandare

**Affiliations:** *Department of Fisheries, Faculty of Fisheries and Environmental Sciences, Gorgan University of Agricultural Sciences and Natural Resources, Gorgan, Iran.*

**Keywords:** Artemia, *Fenneropenaeus indicus*, Intestinal microbiota, Prebiotic, Survival

## Abstract

The present study investigates the effects of different routes of inulin administration as prebiotic on gut microbiota and survival rate of Indian white shrimp post-larvae. Four hundred and fifty Indian white shrimp post-larvae (PL_1_) were stocked in nine tanks. The tanks were assigned into three treatments: feeding with inulin-treated (110 mg L^-1^) *Artemia* nauplii (I-T), feeding with inulin-enriched (110 mg L^-1^) *Artemia* nauplii (I-E) and control which repeated triplicates. Feeding trial was performed until PL_11_ stage and then gut microbiota was studied using culture based method. Also, survival rate was calculated at the end of feeding trial. Our results showed that feeding on inulin enriched or treated *Artemia* nauplii had no significant effect on total viable culturable autochthonous bacteria and *Vibrio* spp. levels of the gut microbiota (*p* > 0.05). However, a remarkable increase of lactic acid bacteria levels (LAB) was observed in I-E treatment (*p* < 0.05). Administration of inulin enriched *Artemia* nauplii significantly elevated survival rates of Indian white shrimp post-larvae (*p* < 0.05). These results encourage administration of prebiotic-enriched *Artemia* nauplii in post larval stage of Indian white shrimp but determination the mode of action of prebiotic on various aspects of shrimp larviculture merit further research.

## Introduction

Shrimp farming accounts for 55% of world shrimp production and during the past decade global shrimp production has rapidly increased to meet market demand.^1^ Despite this rapid growth, shrimp industry has faced with issues raised by infectious viral, bacterial and fungal diseases.^2^ Administrations of antibiotics have been traditionally practiced in shrimp farms at sub-therapeutic levels for disease prevention.^3^ However, emergence of antibiotic-resistant bacteria affected this strategy and nowadays utilization of antibiotics is banned or restricted (European Council Regulation 1831/2003). To resolve the issues raised by antibiotics, administration of environment friendly dietary supplements like probiotics, prebiotics have been suggested. Prebiotics are “non digestible food ingredients which beneficially affect the host by selectively stimulating the growth and/or activity of health-promoting bacteria in the intestinal tract”.^4^ Several studies have demonstrated that prebiotics can improve growth parameters, disease resistance, gut morphology and modulate the intestinal microbiota in various aquatic species.^5^ Inulin and oligofructose are among the most known and well-studied prebiotic in human and terrestrial animals.^6^ Despite some negative results,^7^^-^^10^ several studies have reported positive effects of inulin as growth promoter.^11^ Although numerous studies have been conducted on administration of prebiotics in aquaculture, no information is available on the effects of inulin as prebiotic on shrimp larviculture. To our best knowledge, there is no available information about efficiency of routes of administration of prebiotics in shrimp larvicutlure (i.e. via *Artemia* nauplii treatments or enrichment). 

The present study was designed to determine the changes in gut microbiota and survival rate of Indian white shrimp (*Fenneropenaeus indicus*) post-larvae following administration of inulin enriched and inulin treated *Artemia* nauplii. 

## Materials and Methods

A feeding trial was conducted using Raftiline ST (Raffinerie Tirlemontoise Co., Tienen, Belgium), which is the source of inulin, as prebiotic for shrimp post-larvae. All experiments were conducted at the Abziparavar Chabahar Hatchery (Chabahar, Iran). Indian white shrimp post-larvae were obtained from three eyestalk-ablated spawners. Shrimp nauplii were kept in 100 L spawning tanks containing natural sea water supplemented with a mixture of the microalgae *Chaetoceros* and *Tetraselmis*, which was added daily at a rate of 2 × 10^6^ cells per mL.^12^ The water quality parameters include temperature, salinity, pH and dissolved oxygen maintained at 30.5 ˚C, 37 ppt, 8.10 to 8.20, 6.35 ± 0.22 mg L ^-1^, respectively.

PL_1 _larvae (initial mean length 5.42 ± 0.82 mm) were then transferred to nine plastic tanks (20 L) at a stocking density of 50 larvae L^-1^. The tanks were randomly assigned to three treatments: Control shrimp fed unenriched *Artemia* nauplii (C), shrimp fed inulin-enriched *Artemia* nauplii (I-E) and shrimp fed inulin-treated *Artemia* nauplii (I-T). *Artemia franciscana* cysts (INVE Aquaculture, Dendermonde, Belgium) were hatched according to Sorgeloos *et al*.^13^ by incubating in glass jars at density of 600 mg L^-1^ for 24 hr in saline water (25 ppt) with continuous aeration and light (28.0 ˚C). For the I-E treatment, nauplii were enriched after hatching following Agh and Sorgeloos with minor modifications.^14^ Briefly, newly hatched nauplii were incubated in 500 mL enrichment solution including 150 mg L^-1^ docosahexanoic acid (DHA, INVE Aquaculture, Dendermonde, Belgium) and 60 mg L^-1^ prebiotic powder for 14 hr at 28.0 ˚C. Thereafter, *Artemia* were further enriched with DHA (50 mg L^-1^) and prebiotic powder (50 mg L^-1^) for 12 hr at 28.0 ˚C. For the I-T treatment, decapsulated *A. franciscana* nauplii were hatched in water containing inulin (110 mg L^-1^). Post-larvae were fed at a rate of 8 to 10 nauplii per larva five to six times a day from PL_1_ through 10 days after metamorphosis (PL_11_). Twenty percent of the tank water was exchanged each day. 

All treatments were repeated in triplicate. During the experiments, water temperature, pH and salinity were monitored daily and maintained at 29.10 to 29.90 ˚C, 8.10 to 8.20 and 35 ppt, respectively.

The survival rate of Indian white shrimp post-larvae was calculated at the end of trial according to the following formula: 

$\mathrm{Survival rate}=\frac{Nf}{Ni}\times 100$

where, N_i_ is the initial number of post-larvae and N_f_ is the final number of post-larvae.

At the end of experiment, 20 specimens were sampled randomly from each tank and gut microbiota analysis was performed according to the method previously described by Daniels *et al*.^15^ Briefly, the Indian white shrimp post larva were surface disinfected for 10 min using 0.1% benzalkonium chloride (Merck, Darmstadt, Germany) on ice. Then, all samples (whole body) were rinsed three times in sterilized phosphate-buffered saline and homo-genized with sterile pestles (Bel-Art, Pequannock, US) in sterile 1.5 mL micro-centrifuge tubes. The homogenized sample were then serially diluted with sterile saline (0.85% NaCl) and 100 μL of the samples was spread in triplicate onto three media. Plate count agar (Liofilchem, Roseto degli Abruzzi, Italy), thiosulphate citrate bile salts agar (Oxoid Ltd., Hampshire, UK) and de Man, Rogosa and Sharpe agar (Liofilchem, Roseto degli Abruzzi, Italy) media were used for the enumeration of total viable aerobic heterotrophic bacteria, *Vibrio* spp. and lactic acid bacteria (LAB), respectively. The colony forming units (CFU) per g were calculated from statistically viable plates (i.e. plates containing 30 to 300 colonies).^16^

All statistical analyses were conducted using SPSS (version 10.0; SPSS Inc., Chicago, USA). After checking for normality and homogeneity of variance, data were subjected to a one-way analysis of variance (ANOVA). When significant differences were observed, Duncan's multiple range tests were performed. Mean values were considered significantly different at *p* < 0.05. Data are expressed as mean ± standard error.

## Results

**Fig. 1:**
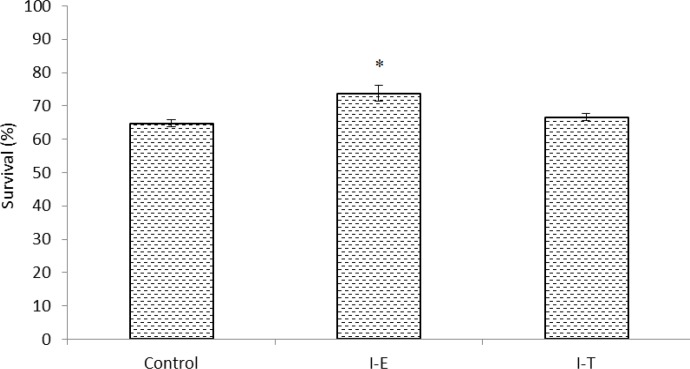
Survival of Indian white shrimp gut microbiota fed un-enriched *Artemia* nauplii (Control) or inulin enriched (I-E) or inulin treated *Artemia* nauplii (I-T). Values are presented as mean ± standard error.

The results of survival rates of Indian white shrimp post-larvae survival rate at the end of feeding trial are presented in Fig. 1. The results showed no significant difference between survival rates of shrimp fed inulin treated *Artemia* nauplii and control group (*p* > 0.05). However, it was significantly elevated in I-E treatment compared to control and I-T treatment (*p* < 0.05). 

**Fig. 2:**
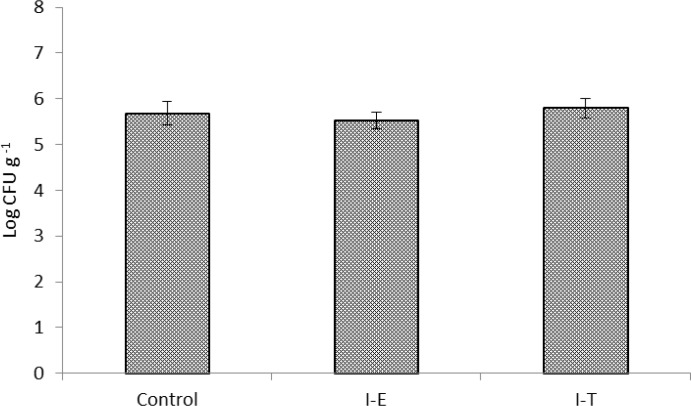
Total culturable autochthonous bacterial levels (log CFU g^-1^) of Indian white shrimp gut microbiota fed un-enriched *Artemia* nauplii (Control) or inulin enriched (I-E) or inulin treated *Artemia* nauplii (I-T). Values are presented as mean ± standard error.

Figure 2 represents total heterotrophic autochthonous bacterial levels in gut microbiota of shrimp post-larvae. Compared to the control group, total heterotrophic autochthonous bacterial levels in shrimp post-larvae fed inulin treated or enriched *Artemia* nauplii were not significantly higher (*p* > 0.05). Also, no significant differences were observed between I-T and I-E treatment in case of total heterotrophic autochthonous bacterial levels (*p* > 0.05). 

**Fig. 3:**
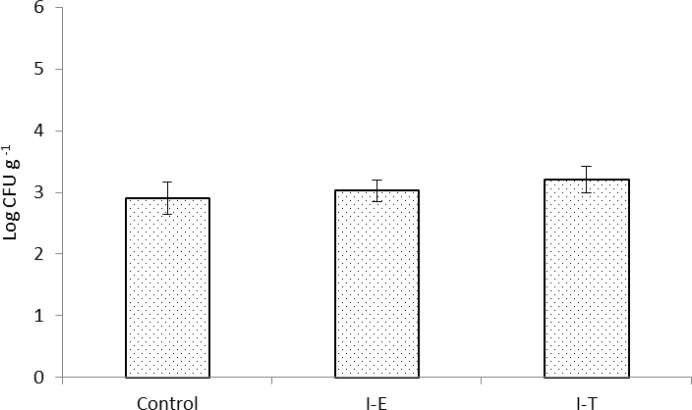
*Vibrio* spp. levels (log CFU g^-1^) in gut microbiota Indian white shrimp fed un-enriched *Artemia* nauplii (Control) or inulin enriched (I-E) or inulin treated *Artemia* nauplii (I-T). Values are presented as mean ± standard error.

**Fig. 4:**
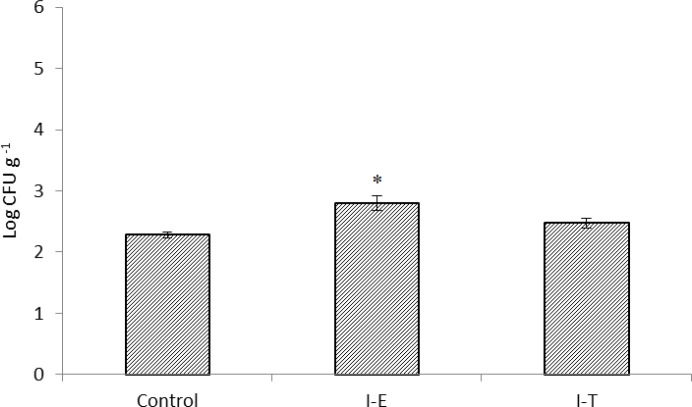
Lactic acid bacteria levels (log CFU g^-1^) in gut microbiota Indian white shrimp fed un-enriched *Artemia* nauplii (Control) or inulin enriched (I-E) or inulin treated *Artemia* nauplii (I-T). Values are presented as mean ± standard error.

Similar to the results obtained in total heterotrophic autochthonous bacterial levels, *Vibrio* spp. levels were not affected by feeding on enriched or treated *Artemia* nauplii and control treatment (*p* > 0.05), (Fig. 3). As shown in Figure 4 culturable LAB levels were significantly increased after 10 days feeding on inulin enriched *Artemia* nauplii ccompared to the control group (*p *< 0.05), (Fig. 4). However, although LAB levels were elevated in I-T, no significant difference was observed when compared to shrimps in control group (*p* > 0.05).

* Asterisk denotes significant difference compare to the other groups (*p* > 0.05).

There are no significant differences among the groups (*p* > 0.05).

There are no significant differences among the groups (*p* > 0.05).

* Asterisk denotes significant difference compare to the other groups (*p* > 0.05).

## Discussion

To our knowledge, there is no published data about the effects of inulin as prebiotic on gut microbiota of Indian white shrimp post-larvae. Only a few studies have reported the effects of different prebiotics on shrimp growth and survival compared to studies performed on fish.^5^ Determination of the effects of potentially beneficial dietary supplements like prebiotics in early stages of life is of high importance both in fish and shrimp as these stages are generally considered as sensitive period.^11^^,^^17^^,^^18^ In the present study, we investigated the effects of feeding inulin to Indian white shrimp post-larvae via *Artemia* nauplii enrichment or treatment (i.e. different routes of administration). The results of the present study showed that post-larvae fed inulin-enriched *Artemia nauplii* (I-E) displayed significantly higher survival compared to both the control and I-T groups. In line with our results, Li *et al*.^19^ reported that feeding Pacific white shrimp (*Litopenaeus vannamei*) post-larvae with 20 g kg^–1^ prebiotic (Grobiotic-A^®^) increased survival rate. Likewise, administration of dietary mannanoligosaccharide (MOS) as prebiotic, increased survival rates in Pacific white shrimp^20^ and Tiger shrimp (*Penaeus semisulcatus*).^21^ However, there are a number of reports indicating prebiotic failed to improve survival of western king prawn (*Penaeus latisulcatus*) juveniles^22^ and Pacific white shrimp juveniles.^23^^,^^24^ The contradictory nature of the results from prebiotic studies conducted with aquatic animals thus far is likely due to the differing methods of prebiotic administration, dosage levels, fermentability of the prebiotics and the different intestinal morphology and microbiota. 

In spite of several studies on probiotics and prebiotic effects on growth performance, there is limited information available on modulation of shellfish gastrointestinal tract microbiota by using prebiotics.^5^ The results of this study showed that feeding on prebiotic enriched and prebiotic treated *Artemia* nauplii, had no significant effects on total heterotrophic autochthonous bacterial and *Vibrio* spp. levels. However, significant increase of LAB levels observed following feeding Indian white shrimp post-larvae with prebiotic enriched *Artemia* nauplii. Elevation of LAB in gut microbiota can be attributed to provision of substrate for growth of these bacteria group (i.e. LAB). It has been well-documented that modulation of gastro-intestinal microbiota toward potentially beneficial communities can be achieved by dietary administration of prebiotics.^11^ In accordance with the findings of this study, Daniels *et al*.^15^ stated that although dietary MOS had no significant effects on *Vibrio* spp., it increased the stability of bacterial populations in the gastrointestinal tract of larval European lobster (*Homarus gammarus*). However, MOS significantly increased cultivable gastrointestinal tract total aerobic bacteria and *Vibrio* spp. levels in tropical spiny lobster (*Panulirus ornatus*) juveniles.^25^ The LAB are considered generally as potentially beneficial communities of gut microbiota which can affect host health. The elevation of survival rate of inulin fed Indian white shrimp post-larvae following feeding on prebiotic occurred possibly due to improving general health and resistance of post-larvae. However, determination of the exact mode of action of prebiotic on shrimp post-larvae merits further research.

In conclusion, this preliminary study suggests promising effects of prebiotic on shrimp post-larvae survival and gut microbiota. The current study warrants further investigations to determine the optimum dosage and administration of prebiotics in shrimp larvae and post-larvae culture. 
